# Highly Integrated MEMS Magnetic Sensor Based on GMI Effect of Amorphous Wire

**DOI:** 10.3390/mi10040237

**Published:** 2019-04-08

**Authors:** Jiawen Chen, Jianhua Li, Lixin Xu

**Affiliations:** School of Mechatronical Engineering, Beijing Institute of Technology, Beijing 100081, China; 13718925459@163.com (J.C.); lxxu@bit.edu.cn (L.X.)

**Keywords:** MEMS, amorphous wire, GMI magnetic sensor, high integrated

## Abstract

In this paper, a highly integrated amorphous wire Giant magneto-impedance (GMI) magnetic sensor using micro electron mechanical system (MEMS) technology is designed, which is equipped with a signal conditioning circuit and uses a data acquisition card to convert the output signal of the circuit into a digital signal. The structure and package of the sensor are introduced. The sensor sensing principle and signal conditioning circuit are analyzed. The output of the sensor is tested, calibrated, and the relationship between the GMI effect of the amorphous wire and the excitation current frequency is explored. The sensor supplies voltage is ±5 V, and the excitation signal is a square wave signal with a frequency of 60 MHz and an amplitude of 1.2 V generated by the quartz crystal. The sensor has the largest GMI effect at 60 MHz with a sensitivity of 4.8 V/Oe and a resolution of 40 nT.

## 1. Introduction

Since Mohri et al. found the Giant magneto-impedance (GMI) effect with its large sensitivity of the electrical impedance in amorphous materials, the continuous study of developing new materials and special treatments to design GMI sensors has become one of intensive research in the field of weak magnetic measurements [1,2,3,4]. Amorphous GMI sensors find a wide range of applications in areas such as security electronics, recording heads, and recently, life science [5]. Recently, the study on enhanced sensitivity, improved signal-to-noise ratio, and the smaller size of amorphous GMI sensors, especially amorphous wire GMI sensors, has provoked great interest due to its wide possibilities and applications [6,7]. Sergey Gudoshnikov et al. designed a magnetometer based on the off-diagonal GMI effect in Co-rich glass-coated amorphous wire [8]. The sensing element of the magnetometer is a 10 mm long piece of CoFeNiBSiMo microwire with a small pick-up coil of 85 turns wound around the microwire. The magnetometer is capable of measuring a narrow range of magnetic fields of ±3.5 µT, with a range of up to ±250 µT when using the feedback circuit. The sensitivity of magnetometer is 30 V/T at I = 0.22 mA. Nikolai A. Usov et al. designed an off-diagonal sensor by utilizing a complex waveform excitation produced by a microcontroller and applied to a multiple wire MI element [5]. Three wires connected in parallel were used as a sensing element and the detection coil had 40 turns of copper wire with a diameter of 60 μm. By optimizing the pulse characteristics, a resolution of 60 mV/Oe was realized with linearity in the field interval of ±3.5 Oe. Nikolay A. Yudanov et al. investigated the off-diagonal impedance of a number of Co-rich glass-coated amorphous wires placed in the same coil [9]. The wires are connected in parallel and excited by a harmonic current with optimized direct current (DC) bias, which is used to remove the domain structure. The maximum sensitivity for two wires sensor is about 380 mV/Oe within the field interval ±1.8 Oe. Shuangcheng Wei et al. designed a magnetic impedance (MI) element with a special structure [10]. The detector was at a low power consumption with a simple circuit structure．It also had a wide linear range of ±3 Oe and high magnetic sensitivity of 65 mV/Oe．Xinhua Nie et al. developed a differential-type integrating GMI magnetic sensor [11]. The offset coil of the sensor is made up of a 0.20 mm diameter enameled wire wrapping for about 200 turns around the amorphous wire. In order to reduce the small stress effect on the sensor, the amorphous wires are fixed on the printed circuit board (PCB) by elastic silicon rubber. The GMI magnetic sensor exhibits a linearity error of about 0.92% in the measuring range of ±2.0 Oe, and the sensitivity can achieve about 748 mV/Oe. Héctor García-Miquel et al. established a general model of field sensor based on a two microwires combination according to the responses obtained [12]. The device provides good linearity and high sensitivity in low field region and other ranges if the field addition in bias coils is used. Without the necessity of amplifying, 45.4 mV/Oe sensitivity in ±1.63 Oe with a maximum linearity error of 0.98%, was obtained. Daniil Karnaushenko et al. realized arrays of GMI sensors, which are directly on-chip integratable in a complementary metal oxide semiconductor (CMOS) compatible process [13]. They put forth a new platform that is relying on photo-patternable, thermally, and chemically stable imide- and acrylic-based polymers allowing for the stimuli-controlled self-assembly of initially planar NiFe-/Cu/NiFe-based heterostructures into 3D tubular architectures possessing the GMI functionality. The sensitivity of the sensor at the excitation frequency of 75 MHz is 45 µV/Oe (zero-field pick-up voltage is 6 mV). Kaneo Mohri et al. developed an amorphous wire CMOS integrated circuit (IC) multi-vibrator type micro-linear magnetic field sensor for industrial usage MI sensor chip [14]. The CMOS IC inverter multi-vibrator was adopted as a pulse train generator for amorphous wire magnetization via a differentiation circuitry and two times delay inverters. The pulse magnetization magneto-impedance gain is adjustable by adjusting the parameter of the differentiating circuitry. The dynamic range of the sensor is ±4 G, the sensitivity is 500 mV/G. The industrial mass production type magnetoimpedance element is fabricated by Aichi Steel Corporation and its size is 0.6 mm × 0.3 mm. In an earlier article, we proposed a GMI magnetic sensor based on amorphous wire produced by the micro electron mechanical system (MEMS) process [15]. The magnetic sensor measures only 5.6 mm × 1.5 mm × 1.1 mm and has a sensitivity of 130 mV/Oe without a signal processing circuit. It can detect a magnetic field range of 0–0.45 Oe. E. V. Golubeva et al. investigated the magnetic properties and specific features of GMI in amorphous rapid-quenched wires of composition (Co0.94Fe0.06)72.5Si12.5B15 [16]. The working interval and sensitivity have been calculated for the alternating current frequencies from 1 MHz to 1 GHz. The magnetic sensor using this kind of amorphous wire as a sensitive material has a maximum sensitivity of 23 %/Oe in the range of 100 MHz to 300 MHz. Yulong Chen et al. presented a novel fabrication method for amorphous alloy wire GMI magnetic sensor based on MEMS technology [17]. In this process, negative SU-8 thick photoresist was proposed as the solder mask due to its excellent properties and the low melting temperature solder paste was used for the electrical connections with the amorphous alloy wire and the electrode pads. The sensor sensitivity is around 150 mV/Oe and the nonlinearity is less than 0.92% F.S. The above various types of magnetic field sensors are summarized in Table 1. 

Although many studies on various aspects of magnetic sensors have been performed, few reports focus on the high resolution and high integration of MEMS magnetic sensors based on amorphous wires. In this paper, the factors affecting the amorphous wire GMI magnetic sensor output are first explored. The structure and the package of the amorphous wire GMI magnetic sensor are introduced. The device and peripheral circuits are designed on a PCB, and the peripheral circuits include a crystal oscillator circuit, a peak detector circuit, a low pass filter circuit, and a DC amplifier circuit. The relationship between the excitation current frequency of the amorphous wire and its GMI effect is also investigated. Finally, the output of the amorphous wire GMI magnetic sensor is tested and the output characteristic curve and sensitivity are obtained. The resolution of the amorphous wire GMI magnetic sensor is 40 nT by the definition of signal-to-noise ratio.

## 2. Sensor Design 

### 2.1. Device Design

The structure of the device is designed as a “loop excitation + non-diagonal extraction” method because the manufacturing process of the structure is simple, and the working conditions of the amorphous wire are easy to determine. When the excitation current is applied to both ends of the amorphous wire, the output voltage of the coil is obtained by the Faraday’s law of electromagnetic induction, as shown in Equation (1).

(1) $$U_{i}=-N\frac{d\Phi }{dt}=-N\frac{dB\times S}{dt}=-NS\mu_{0}(1+\frac{dM}{dH})\frac{dH_{e}}{dt}$$

where *N* is the number of turns of the pick-up coil, Φ is magnetic flux of the pick-up coil, *B* is the magnetic induction of the amorphous wire, *S* is the cross-sectional area of the pick-up coil, *M* is the magnetization of the amorphous wire, *H_e_* is the excitation magnetic field strength generated by the excitation current, *μ_0_* is vacuum permeability. 

It can be seen from Equation (1) that the output voltage of the sensor is related to the size of the pick-up coil, the magnetization curve of the amorphous material, and the magnetic field generated by the excitation current. Therefore, using an amorphous wire with high magnetic permeability and increasing the number of turns and the cross-sectional area of the pick-up coil can improve the output sensitivity of the sensor. According to the Biot-Savart law, the magnetic field generated by the excitation current is related to the magnitude and frequency of the current, thus the output voltage of the sensor is also related to the magnitude and frequency of the excitation current. 

**Figure 1:**
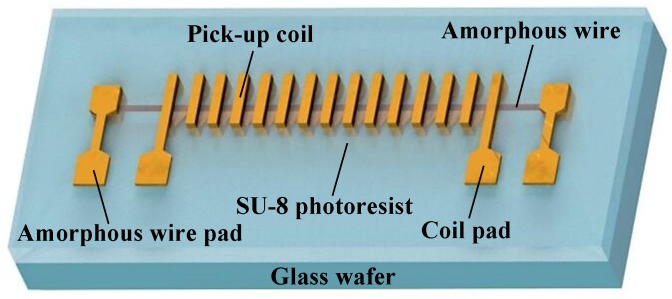
Schematic diagram of the amorphous wire Giant magneto-impedance (GMI) magnetic sensor device. It can be seen from Equation (1) that in order to improve the sensitivity of the sensor, the turns of the micro-coil, the height of the micro-coil pillar, and the length of the micro-coil wire can be increased.

In order to obtain an amorphous GMI magnetic sensor with small size and high device uniformity, a square pick-up coil was designed and integrated with the amorphous wires using the MEMS processes [15]. The new structure makes it easier and more flexible to change the geometry of the pick-up coil, thus allowing more precise control and change of the output voltage of the amorphous GMI magnetic sensor. The device structure is shown in Figure 1. 

**Figure 2:**
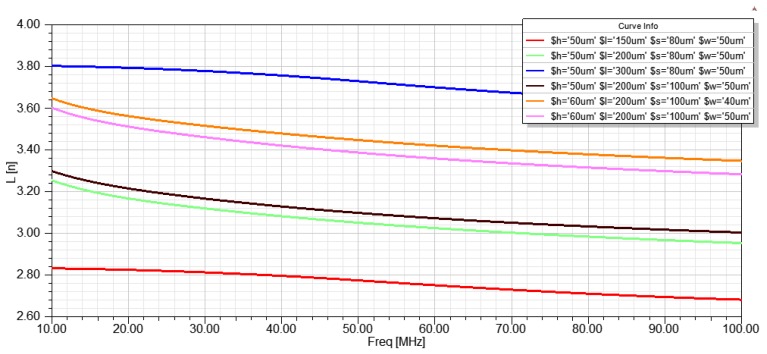
The influence of the geometric parameters of the microstructure coil on the inductance value. It can be seen from the results that the inductance value of the micro-coil is positively correlated with the length *l* of the coil wire and is negatively correlated with the space *s* of each turn of the coil.

The glass wafer serves as a device support layer, and copper is electroplated on the glass wafer to form a microstructure pick-up coil. The microstructure coil has a square cross section with an amorphous filament placed longitudinally at its center. The geometric parameters of the microstructure pick-up coil are given by the simulation results of the High Frequency Structure Simulator (HFSS) software. It can be seen from Equation (2) that the induced electromotive force of the micro-coil is proportional to the inductance of the micro-coil [15]:

(2) $$E_{c}=NL_{c}\frac{dI_{c}}{dt}\;$$

where *E_c_* is the induced electromotive force of the micro-coil, *N* is the turns of the micro-coil, *L_c_* is the micro-coil inductance, *I_c_* is the induced current across the micro-coil. By establishing a microstructure inductive coil model in HFSS and obtaining the relationship between the inductance value and the geometric parameters of the micro-coil, the optimal parameters of the micro-coil can be determined. The simulation results are shown in Figure 2, and the turns of the pick-up coil are 10. 

The composition of the selected amorphous wire is CoFeSiB. Other parameters of the amorphous wire are shown in Table 2. 

**Figure 3:**
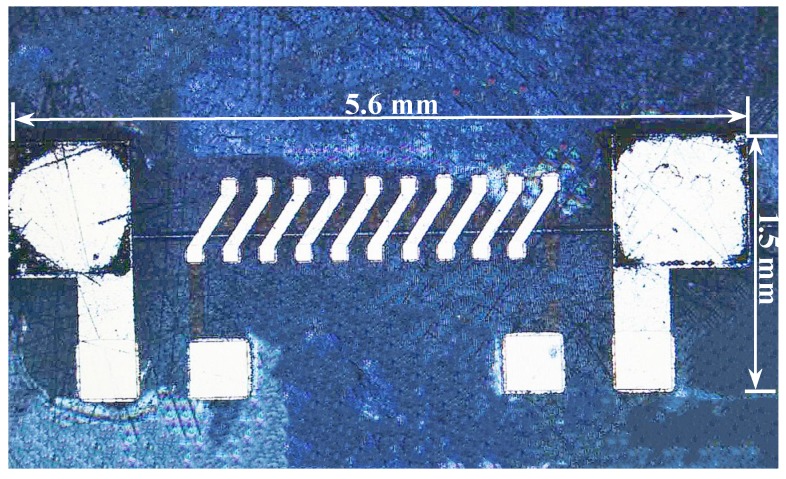
The electron micrograph of the amorphous GMI magnetic sensor. The size of the magnetic sensor is 5.6 mm × 1.5 mm × 1.1 mm.

The amorphous wire is fixed by copper electroplated at both ends thereof and attached to the Cr/Cu seed layer. Except for the amorphous wire and the pick-up coil pad, the entire device is wrapped with a SU-8 photoresist to avoid deformation and fracture of the amorphous wire and the micro-coil. The resulting amorphous GMI magnetic sensor device has a size of 5.6 mm × 1.5 mm × 1.1 mm, which reduces the sensor volume compared to the method of soldering and manual winding and improves the device uniformity of the sensor. The electron micrograph of the amorphous GMI magnetic sensor device is shown in Figure 3.

**Figure 4:**
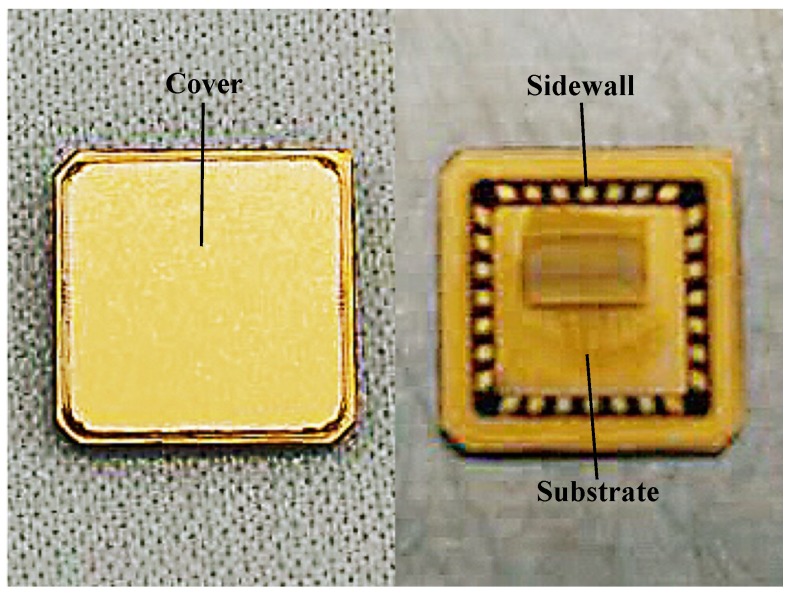
Silicon nitride ceramic package of the amorphous GMI magnetic sensor device. The package includes a substrate, a sidewall, and a cover.

To further protect the microstructure of the amorphous GMI magnetic sensor device and facilitate the integration of sensor circuits, a silicon nitride ceramic tube was used as a package for the device, as shown in Figure 4. The ceramic package can form a hollow, airtight structure with excellent mechanical rigidity, and the thermal expansion coefficient is close to that of silicon, thus the stress with the silicon substrate is small. The silicon nitride ceramic package includes a substrate, a sidewall, and a cover. The substrate is fixed to the amorphous GMI magnetic sensor device by welding. The inner and outer sides of the sidewall are provided with pads and electrically connected, and the amorphous wires and the coil pads are respectively connected to the ceramic package pads by wire bonding techniques. The cover plate is fixed on the upper surface of the sidewall of the ceramic tube by adhesive to form a sealing structure, and the inside of the tube is filled with nitrogen to form an airtight environment.

### 2.2. Circuit Design

**Figure 5:**
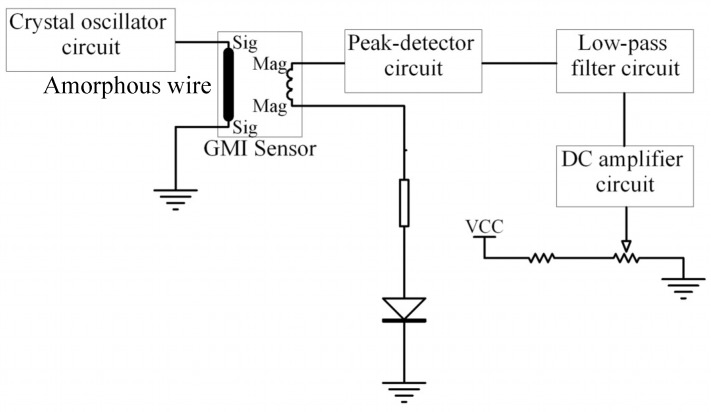
Sensor circuit block diagram. The circuit realizes the function of converting a magnetic field signal into a direct current (DC) voltage signal.

In order to preprocess the output voltage of the amorphous wire GMI magnetic sensor device, a signal conditioning circuit was designed. Most current GMI magnetic sensors use a CMOS multi-vibrator to generate the excitation current and an analog switch as the peak sample-and-hold circuit, as proposed by Professor Mohri [14]. These circuits are complex in structure and poor in flexibility. We only used the crystal oscillator circuit and an improved diode detector circuit to ensure the high performance of the sensor without the feedback circuit, which helped to ensure the consistency of the amorphous GMI magnetic sensor in the productization. The sensor system was composed of the amorphous wire device, a crystal oscillator circuit, a peak-detector circuit, a low-pass filter circuit, and a DC amplifier circuit, as shown in Figure 5. 

**Figure 6:**
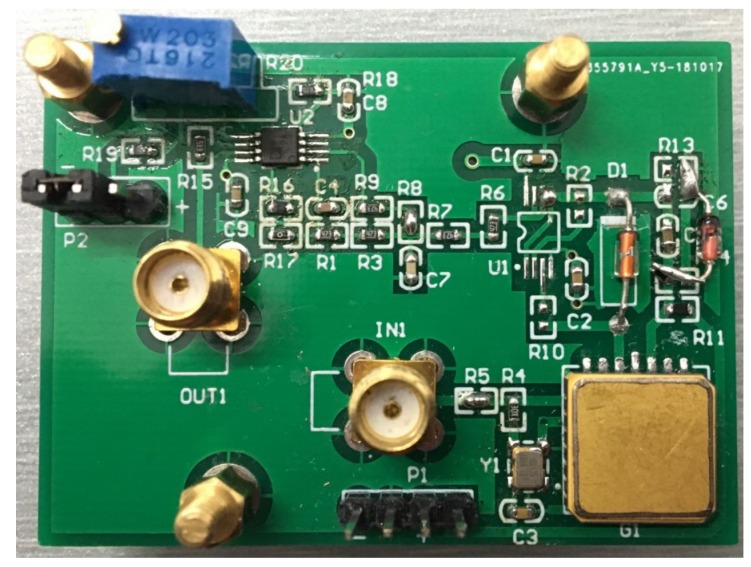
The amorphous wire GMI magnetic sensor circuit printed circuit board (PCB). The device of the amorphous GMI magnetic sensor is packaged in a silicon nitride ceramic tube and soldered on the PCB.

Since the excitation signal generated by the crystal oscillator is stable in frequency and simple in circuit and low in cost, 1.2 V_pp_ quartz crystal oscillator was selected as a signal generating circuit to provide high frequency square wave excitation for the amorphous wire. The improved diode detection circuit is used to demodulate the magnetic field signal in the output signal of the amorphous wire GMI magnetic sensor. We have added two operational amplifiers before and after the diode to improve the nonlinearity of the diode and eliminate the dead zone of the diode. The detection circuit consists mainly of a Schottky diode, a capacitor, two resistor, and two follower amplifiers with high input impedance. The equivalent turn-on voltage of the diode is:

(3) $$U_{i}=U_{D}/A_{d}$$

where *A_d_* is the open-loop gain of the operational amplifier, generally above 80 dB; *U_D_* is the diode deadband voltage. For Schottky diodes, *U_D_* ≈ 0.2 V. *U_i_* is very small, approximately zero, which eliminates the dead zone of the diode. At the same time, the operational amplifier operates in the voltage follow state when the diode is turned on, thereby eliminating the nonlinear effects of the diode. Some high-frequency components in the detection signal cannot be completely attenuated due to the limitation of the detection circuit time constant, and the interference of the external magnetic field cannot completely eliminate the ringing phenomenon, thus the detection signal needs to be smoothed before the amplification circuit. Compared to other low-pass active filters, the Butterworth filter has the most stable amplitude characteristics in the passband and good linear phase characteristics. The amorphous magnetic sensors mainly focus on the amplitude-frequency characteristics of the filter circuit; thus, a second-order Butterworth low-pass filter is used to achieve signal filtering. According to the magnetic field frequency characteristics of the target to be detected and the requirements of filtering the carrier wave and the high frequency interference signal, the cutoff frequency of the low pass filter is determined to be 1 kHz, and the resistance value and the capacitance value in the filter circuit can be determined by the transfer function. In order to minimize the sensor size, we use the same OP2177 chip to realize the DC amplifier circuit and the low-pass filter circuit. The OP2177 is a dual low-noise operational amplifier with extremely low offset voltage characteristics and input bias current characteristics, thus ensuring low noise introduced. Since the giant magneto-impedance effect of the sensor is very obvious, the voltage output value changes greatly within the selected range, thus the magnification is selected as 20. In addition, a sliding rheostat is connected to the forward input of the op amp to ensure that the magnetic sensor outputs at a zero value at zero magnetic field. The sensor system is powered by a ±5 V dual supply, and the entire amorphous wire GMI magnetic sensor PCB board is shown in Figure 6.

## 3. Experiment

### 3.1. Excitation Frequency Test

The frequency of the excitation current strongly influences the GMI effect, and the amorphous wire will exhibit distinct characteristics in different frequency ranges. The current frequency of research on GMI materials has reached several GHz [18,19,20,21]. In order to explore the optimum operating frequency of the amorphous wire material we used, the detection circuit was designed, which included a digital signal generator, the amorphous wire, and a standard resistor. The digital signal generator supplied the amorphous wire with current excitation of different frequencies. The current signal waveform is a sine wave with an amplitude of 5 V_pp_ and an offset of 2.5 V. The amorphous wire was connected in series with the standard resistor to obtain the current. The entire circuit was placed in an FE-1MF Helmholtz coil that provided an applied magnetic field, and an oscilloscope was used to measure the peak value of the output voltage across the standard resistor. It is worth noting that in order to avoid the influence of the earth’s magnetic field, the applied magnetic field *H_ex_* is always perpendicular to the direction of the earth’s magnetic field during the measurement. Limited by the experimental conditions, we calculated the resistance of the amorphous wire instead of the impedance to characterize the GMI effect of the amorphous wire, resulting in Equation (4).

(4) $$GMI(\%)=\frac{\Delta R}{R}(\%)=\frac{R(H_{ex})-R(H_{max})}{R(H_{max})}\times 100\%$$

where *R*(*H_ex_*) and *R*(*H_max_*) represent the alternating current resistance of the amorphous wire when the external magnetic field is an arbitrary value and a saturated state, respectively. According to the voltage division principle of the series circuit, *R*(*H_ex_*) and *R*(*H_max_*) can be represented by the voltage values measured in the circuit, as shown in Equation (5) and (6).

(5) $$R(H_{ex})=R_{0}\times \frac{U_{2}}{U_{1}}$$

(6) $$R(H_{max})=R_{0}\times \frac{U_{2}^{\prime }}{U_{1}^{\prime }}$$

where *R_0_* is the standard resistor, *U_2_* and *U_1_* are the voltage of the amorphous wire and the standard resistor at arbitrary magnetic field, *U_2_’* and *U_1_’* are the voltage of the amorphous wire and the standard resistor at saturated magnetic field, respectively. From Equations (4), (5), and (6), the expression of the GMI effect can be written as Equation (7).

(7) $$GMI(\%)=\frac{R(H_{ex})}{R(H_{max})}-1=\frac{U_{2}}{U_{1}}\times \frac{U_{1}^{\prime }}{U_{2}^{\prime }}-1$$

**Figure 7:**
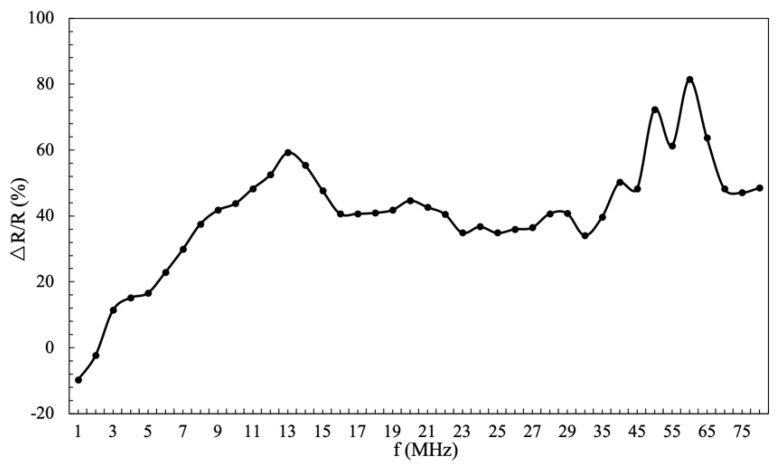
The curve of the amorphous wire GMI effect with the excitation frequency.

Considering that the internal resistance of the Agilent 33220A signal source is 50 Ω and the amorphous wire resistance is 12 Ω from the previous article [15], the standard resistance was selected to be 100 Ω. Thus, the relationship between the GMI effect of the amorphous wire and the excitation current frequency was obtained, as shown in Figure 7. It can be seen from the result that the amorphous wire had the largest impedance change rate when the driving frequency was 60 MHz, thus the crystal oscillator circuit should be selected as 60 MHz.

### 3.2. Output Test

Hardware devices used for testing and calibration of amorphous wire GMI magnetic sensor include voltage sources, Helmholtz coils, data acquisition cards, and a computer. The data acquisition card is NI USB-4431, which has 4 analog input channels (voltage range ±10 V) and a single analog output channel (voltage range ±3.5 V) with a resolution of 24 bits and a sampling rate of up to 51.2 KS/s per channel. The data acquisition card can achieve a good signal-to-noise ratio for analog output (AO) and analog input (AI) channels. A visualization program was written in LabVIEW to observe the change trend of the collected voltage through the program front panel, and the data such as the DC effective value, the maximum value, and the minimum value of the sampling result are automatically saved in Excel, which is convenient for reading and calculating in the computer. When collecting the voltage signal, place the amorphous wire GMI magnetic sensor in the middle of the Helmholtz coil, set the sampling rate of the data acquisition card to 51.2 KHz, and gradually increase the Helmholtz coil magnetic field from the forward and reverse stroke of −0.5 Oe to 0.5 Oe.

**Figure 8:**
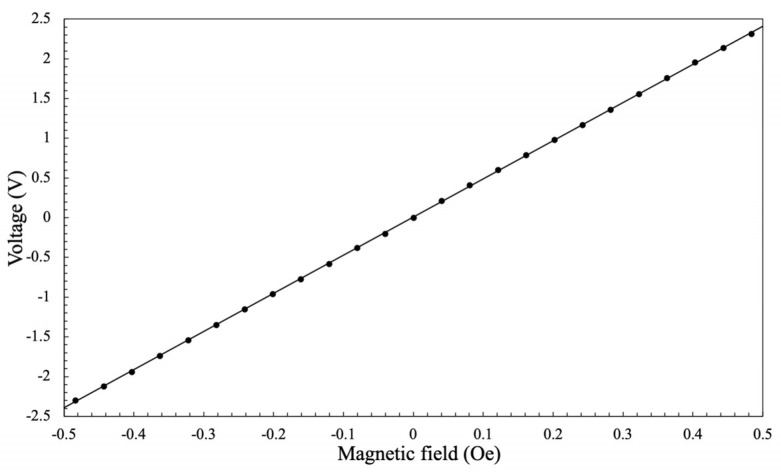
The input and output characteristic curves of the amorphous wire GMI magnetic sensor.

The test results were fitted by the least squares method, and the input and output characteristic curves of the amorphous wire GMI magnetic sensor are shown in Figure 8. 

The fitting equation is Equation (8).

(8) $$V_{out}=4.8011H_{ex}+0.0085$$

where *V_out_* represents the output voltage of the amorphous GMI magnetic sensor. In Equation (8), the unit of the output voltage is V, and the unit of the applied magnetic field is Oe. By analyzing the measurement results of the calibration test, it can be concluded that the full-scale output of the amorphous wire GMI magnetic sensor *Y_FS_* is 5 V. Sensitivity is represented by *K* and its magnitude is equal to the maximum slope of the sensor’s input and output characteristic curves, as shown in Equation (9).

(9) $$K=\left|\frac{\Delta V_{f}}{\Delta H_{ex}}\right|=4.8\;V/Oe$$

The resolution of the amorphous wire GMI magnetic sensor can be defined by the signal-to-noise ratio (SNR). This shows how small an increment in output signal caused by an external field can be before it is indistinguishable from the background noise of the sensor. For the amorphous wire GMI magnetic sensor, the *Y_FS_* is 5 V, and the corresponding range is −0.5 Oe to 0.5 Oe. The oscilloscope observes a peak-to-peak output voltage of 2 mV, which is a noise level of 2 mV. Therefore, the resolution of the sensor is the magnetic field strength corresponding to 2 mV, which is calculated to be 40 nT. The sensitivity of the amorphous GMI magnetic sensor far exceeds the magnetic sensor products listed in Table 1, and the detection accuracy of the magnetic field is increased to the Natsra level. Therefore, the amorphous GMI magnetic sensor can play a huge role in the field of high-precision weak magnetic detection.

## 4. Conclusions

In this paper, a micro-structured amorphous GMI magnetic sensor was designed and fabricated using a MEMS process that precisely controls the amorphous GMI magnetic sensor output by changing the micro-coil parameters. A simple and efficient signal conditioning circuit was designed and integrated with the device on the PCB. The size of the amorphous wire GMI magnetic sensor device is 5.6 mm × 1.5 mm × 1.1 mm, wherein the co-based amorphous wire has a length of 5 mm and the number of pick-up coil turns is 10. The optimum excitation current frequency of the amorphous wire GMI magnetic sensor is 60 MHz. Under this frequency, the GMI sensor has the sensitivity of 4.8 V/Oe and the resolution of 40 nT and can measure the weak magnetic field in the range of −0.5 Oe to 0.5 Oe. The amorphous GMI magnetic sensor achieves high integration and high resolution using the most concise MEMS sacrificial layer process and simple signal conditioning circuitry, saving manufacturing cost and time while ensuring miniaturization of the magnetic sensor and device uniformity, which can provide inspiration for mass production of high precision GMI magnetic sensors in the future.

## Figures and Tables

**Table 1:** Summary of different types of magnetic field sensors.

Type	Fabrication Method	Sensitivity	Range
Co-based off-diagonal GMI sensor	Welding and winding	30 V/T	±250 µT
Fe-based off-diagonal MI Sensor	Welding and winding	60 mV/Oe	±3.5 Oe
Off-diagonal MI sensor with double amorphous wires	Welding and winding	380 mV/Oe	±3.8 Oe
Magnetic detector with conducting layer	Welding and winding	65 mV/Oe	±3 Oe
Differential-type integrating GMI magnetic sensor	Welding and winding	748 mV/Oe	±2 Oe
GMI magnetic sensor with double microwires	artificial winding	0.57 mV/(A/m)	±130 A/m
On-Chip-Integrated GMI sensor	MEMS	45 µV/Oe	±2 Oe
Amorphous wire CMOS IC MI sensor	MEMS	500 mV/G	±4 G
MEMS electrical contact amorphous magnetic sensor	MEMS	150 mV/Oe	±1 Oe

**Table 2:** The Parameters values of the amorphous wire.

Parameters of the Amorphous Wire	Value
Length, *l_a_*	5 mm
Diameter, *d*	75 μm
Resistivity, *R_a_*	110 μΩ/cm
Saturation magnetic induction, *B_s_*	0.7 T
Saturation magnetostriction coefficient, *λ_s_*	−0.065 ppm
Residual magnetic induction, *B_r_*	0.4 T
Crystallization temperature, *T_c_*	240 °C Curie temperature
Coercive force, *F*	5 A/m
